# Analysis of parental origin of *de novo* pathogenic CNVs in patients with intellectual disability

**DOI:** 10.1590/1678-4685-GMB-2023-0313

**Published:** 2024-08-09

**Authors:** Samara Socorro Silva Pereira, Irene Plaza Pinto, Victor Cortázio do Prado Santos, Rafael Carneiro Silva, Emília Oliveira Alves Costa, Alex Silva da Cruz, Aparecido Divino da Cruz, Cláudio Carlos da Silva, Lysa Bernardes Minasi

**Affiliations:** 1 Pontifícia Universidade Católica de Goiás, Escola de Ciências Médicas e da Vida, Programa de Pós-Graduação em Genética, Núcleo de Pesquisa Replicon, Goiânia, GO, Brazil.; 2 Secretaria Estadual de Saúde de Goiás, Centro Estadual de Reabilitação e Readaptação Dr. Henrique Santillo, Goiânia, GO, Brazil.; 3 Universidade Federal de Goiás, Programa de Pós-Graduação em Genética e Biologia Molecular, Goiânia, GO, Brazil.

**Keywords:** CMA, segmental duplication, NAHR, chromosome rearrangement

## Abstract

Chromosomal Microarray Analysis (CMA) has increased the comprehension of the mechanisms of copy number variation (CNV) formation, classification of these rearrangements, type of recurrence, and its origin, and has also been a powerful approach to identifying CNVs in individuals with intellectual disability. The aim of this study was to establish the parental origin of *de novo* pathogenic CNV in a cohort of patients with intellectual disability from the public health system of Goiás-Brazil. CMA was done in 76 trios and we identified 15 *de novo* pathogenic CNVs in 12 patients with intellectual disability. In a total of 15 *de novo* pathogenic CNV, 60% were derived from the maternal germline and 40% from the paternal germline. CNV flanked by low copy repeats (LCR) were identified in 46.7% and most of them were of maternal origin. No significant association was observed between paternal age and the mutation rate of *de novo* CNVs. The presence of high-identity LCRs increases the occurrence of CNV formation mediated by non-allelic homologous recombination and the majority of paternal CNVs are non-recurrent. The mechanism of formation of these CNV may have been by microhomology-mediated break-induced replication or non-homologous end joining.

## Introduction

Chromosomal Microarray Analysis (CMA) has been considered a first-tier approach for neurodevelopmental disorders and/or multiple congenital anomalies diagnosis due to the ability to identify Copy Number Variations (CNVs) associated with human diseases. The 2021 guidelines from the American College of Medical Genetics and Genomics (ACMG) propose exome and genome sequencing (ES/GS) as a first- or second-tier test. In consideration of the substantial cost associated with ES/GS, the recommendation advises conducting CMA or targeted gene sequencing initially, reserving ES/GS for cases were deemed necessary (Manickam et al., 2021; Kim et al., 2023). Identifying the genomic rearrangements underlying a CNV is essential not only to diagnose genetic disorders and provide adequate genetic counseling for the families but also to understand the mechanism of CNV formations and what could contribute to medical outcomes (Schaaf et al., 2011; Cuthbert et al., 2019; Riggs et al., 2020).

CNVs are formed by gain or loss of genomic DNA >50bp and are spread throughout the whole genome, contributing to genetic variation and phenotypic diversity. They are categorized as inherited or *de novo* variations and *de novo* CNVs are the principal cause of intellectual disability, autism spectrum disorder, multiple congenital anomalies, and schizophrenia (Arlt et al., 2012; Stobbe et al., 2014; Zarrei et al., 2015; D’Arrigo et al., 2016). *De novo* CNVs could occur in maternal or paternal germline and during fetal development. During gametogenesis, some mechanisms could be involved in the CNV formation and could emerge all along DNA replication, repair, and chromosome segregation (Ma et al., 2017).

About 4-5% of the human genome consists of segmental duplication, also known as low copy repeats (LCRs), DNA segments from 10 to 400kb in length with 95-98% similarity. Genomic regions with LCRs of high similarity and directly-oriented are hotspots for non-allelic homologous recombination (NAHR), which potentiates the formation of recurrent CNVs that share the same genomic interval occurring in unrelated individuals (Liu et al., 2012; Carvalho and Lupski, 2016). On the other hand, barely is known regarding the molecular mechanism subjacent related to the formation of non-recurrent CNV (Arlt et al., 2012).

The genomic stability of germline cells is essential for the efficiency of reproduction and the proper and healthy development of the fetus. Nonetheless, genomic rearrangements may arise from problems during parental gametogenesis. Due to differences between the gametogenesis of males and females, studies to evaluate the parental contribution of *de novo* CNVs are critical to increasing the biological knowledge regarding the non-recurrent and rare CNVs and their role in the developmental brain in humans (Hehir-Kwa et al., 2011; Ma et al., 2017). In the current study, we identified the parental origin of *de novo* pathogenic CNVs in a cohort of patients with intellectual disability from the public health system of Goiás. 

## Subjects and Methods

### Patient samples

This is a retrospective cross-sectional study from 2013 to 2015, composed of 76 trios who were referred to Replicon Research Group from the Pontifical Catholic University of Goiás and the Laboratory of Human Cytogenetic and Molecular Genetics from State Health Secretary of Goiás as part of a mutual collaborative effort to offer clinical and genetic diagnostic testing for patients with neurodevelopmental delay. All patients included in this study had intellectual disability and were referred with a prior karyotype result without visible numerical or structural chromosomal alteration. The study was approved by the Research Ethics Committee from the Pontifical Catholic University of Goiás, under protocol code 1721/2011. The parents voluntarily signed an informed consent form approved by the Ethics Committee on Human Research. The study was performed under the guidelines of the Declaration of Helsinki.

### SNP array analysis 

Genomic DNA from all 76 probands and their biological parents were isolated from the peripheral blood using Illustra Blood GenomicPrep^®^ Mini Kit (GE Healthcare Life Sciences, USA).

The Chromosomal Microarray Analysis (CMA) was carried out using the GeneChip^®^ CytoScanHD^TM^ (ThermoFisher Scientific, USA), an SNP-array genotyping matrix comprehensive to the human genome of medical interest. The GeneChip^®^ CytoScanHD^TM^ has been recognized for its coverage and ability to communicate with the DGV, OMIM, and RefSeq gene databases. This chip matrix was used for excellent human genome coverage, with 1.9 million non-polymorphic probes combined with 750,000 SNP probes. CMA was carried out according to the manufacturer’s recommendations.

Chromosomal analyses were performed using the Chromosome Analysis Suite 3.0 (ChAS^®^) software (ThermoFisher Scientific, USA) based on the genome reference hg19/GRCh37, using a filter with 50 markers for gains and 25 markers for losses, both with size ≥100 kb. CNVs were classified according to their nature based on previously published international consensus and guidelines (Miller et al., 2010; Kearney et al., 2011; Battaglia et al., 2013; Silva et al., 2019; Riggs et al., 2020). Using the Role Index Score from ChAS software, biological paternity and maternity were confirmed with an index of 99.99%.

### Analysis of parent-of-origin 

The analysis of parental origin was performed using the Mendelian error check function in ChAS^®^ based on SNP variation found in children when compared to each biological parent. The comparison allowed the estimation of the frequency distribution of Mendelian errors in the child, according to the expected Mendelian inheritance of biallelic SNPs from parental genotypes, leading to the calculation of the Mendelian Error Rates (MER). The parental origin of *de novo* pathogenic CNVs was performed using the coordinates of each region analyzed, where the parental chromosome with a low number of errors was considered the chromosome that originated the *de novo* pathogenic CNVs in the child.

### CNV germline mutation rate estimates 

The rate of *de novo* pathogenic CNVs per locus per generation and the estimate of the contribution of the number of paternal meiosis based on the fathers’ age at the time of conception were calculated according to Costa et al. (2018). 

### LCR structure analysis 

Using the Segmental Duplication track of the http://www.genome.ucsc.edu browser (Human Genome reference hg19/GRCh37), we performed an analysis of LCRs with over 90% similarity flanking the *de novo* pathogenic CNVs. CNV coordinates from the patients was used to define the flanking regions, and then a zoom of 3x in CNV size was applied for segmental duplication calling. Genomic rearrangements flanked by LCRs were categorized as recurrent and genomic rearrangements non-flanked by LCRs were categorized as nonrecurrent (Carvalho and Lupski, 2016).

### Statistical analysis 

Using the SPSS® version 21.0 (IBM SPSS Statistics, Armonk, NY, IBM Corp) we performed the statistical analysis. The simple linear regression test was done to observe the influence of paternal age in the mutation rate. All analyses were carried out with a 95% confidence interval (p <0.05).

## Results

After genomic analysis of the 76 patients with ID and their biological parents, we identified *de novo* pathogenic CNVs in 13 patients. MERs were calculated for all patients with pathogenic CNVs. Nevertheless, we could not define the parental origin of the pathogenic CNV from one patient because the MER was very similar between his mother and father. This pathogenic CNV was not included in the final analysis, and the parental origin was reported for 12 patients out of 13 trios analyzed by CMA. Thus, the cohort was composed by 8/12 (66.7%) females and 4/12 (33.3%) males, and the probands’ ages ranged from 1 to 22 years, with an average of 10.2 years. 

**Figure 1-:**
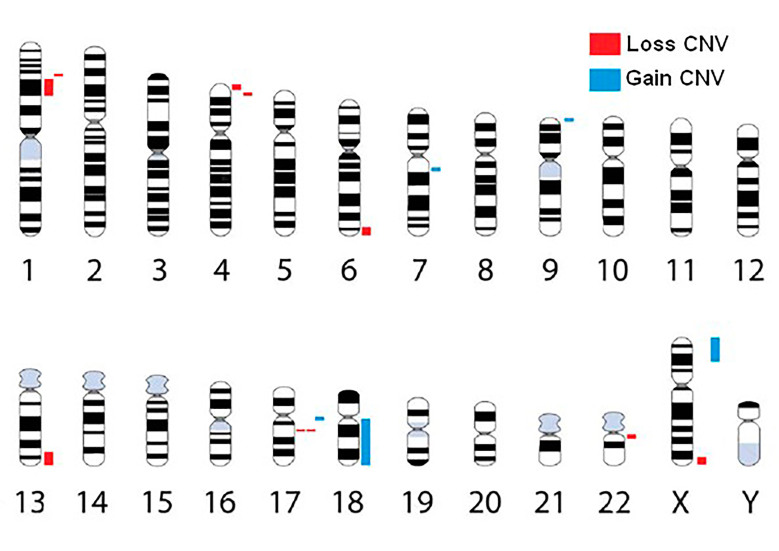
Schematic representation of the human chromosomal ideograms indicating *de novo* pathogenic CNVs in children with intellectual disabilities. The red bands next to the chromosomes represent genomic losses and the blue bands represent genomic gains.

In our group of patients with ID, 15 *de novo* pathogenic CNVs were found in 12 patients, corresponding to 10/15 (66.7%) losses and 5/15 (33.3%) gains. The CNVs distributions were shown in Figure 1.

In our cohort, analyzing the *de novo* pathogenic losses CNVs we observed that 7/10 were maternal origin, whereas 3/10 were paternal origin. On the other hand, 3/5 of *de novo* pathogenic gains CNVs were of paternal origin, and 2/5 were of maternal origin. The CNV size distribution and their parental origin are demonstrated in Table 1.

Analyzing the presence of LCRs flanking the CNVs, we detected 7/15 (46.7%) CNVs flanked by LCRs with ≥ 90% similarity, and 71.4% of CNVs flanked by LCRs were of maternal origin (Table 1). 

**Table 1-:** Clinical and genomic characteristics and parental origin analysis of patients with *de novo* pathogenic CNVs.

Case	Age (yo)	Sex	Clinical features*	Age M**	Age P**	CNV	Microarray Nomenclature	Size (Mb)	Origin	Rearrangement	Similarity of the LCRs (%)
002	11	M	ID	37	25	Gain	46,XY.arr[GRCh37] 17p11.2(16769800_20446820)x3 dn	3.68	Pat	Recurrent	98.8
006	11	F	ID	25	28	Loss	46,XX.arr[GRCh37] Xq27.3q28(144580614_148757072)x1 dn	4.18	Mat	Nonrecurrent	***
007	4	F	GDD, MS, MCA	33	47	Gain	46,XX.arr[GRCh37] 18q11.1q23(18608373_78014123)x2-3 dn	59.41	Pat	Nonrecurrent	***
						Gain	46,XX.arr[GRCh37] Xp22.33p21,3(168546_25887307)x3 dn	25.72	Mat	Recurrent	100
011	8	F	ID,MS,BD	25	29	Loss	46,XX.arr[GRCh37] 1p31.3p31.1(68693129_79580916)x1 dn	10.89	Mat	Recurrent	91.3
013	16	M	ID	15	19	Gain	46,XY.arr[GRCh37] 7q11.23(72718277_74147166)x3 dn	1.43	Pat	Recurrent	99.8
023	1	F	GDD,SS,MS,MCA	31	31	Loss	46,XX.arr[GRCh37] 4p16.3(68345_3926333)x1 dn	3.86	Pat	Nonrecurrent	***
						Loss	46,XX.arr[GRCh37] 4p16.3p16.2(4177795_5724404)x1 dn	1.55	Pat	Nonrecurrent	***
						Gain	46,XX.arr[GRCh37] 9p24.3p24.2(203861_4416073)x3 dn	4.21	Mat	Nonrecurrent	***
026	6	F	GDD, MS,MCA	32	38	Loss	46,XX.arr[GRCh37] 13q32.3q34(99712845_115107733)x1dn	15.39	Pat	Nonrecurrent	***
034	4	M	GDD	29	47	Loss	46,XY.arr[GRCh37] 1p32.3(53894316_55487208)x1 dn	1.59	Mat	Nonrecurrent	***
036	15	M	ID	23	23	Loss	46,XY.arr[GRCh37] 22q11.21(18916842_21800797)x1 dn	2.88	Mat	Recurrent	98.2
042	6	F	ID, M	28	28	Loss	46,XX.arr[GRCh37] 6q26q27(162708065_170919482)x1 dn	8.2	Mat	Nonrecurrent	***
043	18	F	ID	22	26	Loss	46,XX.arr[GRCh37] 17q21.31(43648662_44212416)x1 dn	0.56	Mat	Recurrent	98.3
063	22	F	ID, M,DBEA	32	48	Loss	46,XX.arr[GRCh37] 17q21.31(43703801_44212416)x1 dn	0.51	Mat	Recurrent	98.3

*ID=Intellectual disability; GDD=Global Developmental Delay; M=Microcephaly; MS=Multiple Stigmas; BD=Behavior Disorders; MCA=Multiple Congenital Abnormalities; SS=Short

Stature; DBEA=Disturbance of brain electrical activity;**Age M: Maternal age at conception; Age P: Paternal age at conception;*** Genomic region did not flanked by LCR with over

90% of similarity.

Assessing the parental age at conception, we could observe that the average maternal and paternal age was 27.7 and 32.2 years, respectively. After obtaining the germline mutation rate and estimating the number of paternal meiosis, the simple linear regression test was performed to understand if paternal age influenced the mutation rate of *de novo* pathogenic CNVs. Thus, it was observed that there was a growth in the CNV mutation rate when associated with the father’s age, although it was not statistically significant (R² = 0.65, p> 0.05).

## Discussion

Since 1980, Charmberlin and Magenis (1980) affirmed that the parent’s lifestyle should be investigated to understand the mechanism related to the formation of chromosomal rearrangements, with mutational processes and the contribution of parental ages being important in new genomic rearrangement formation. Male individuals contribute to the formation of genomic rearrangements of their descendants, mostly with point mutations due to the numerous replications during the division of pre-meiotic spermatogonia cells. On the other hand, the female germ cells undergo meiosis during fetal ovarian development, stand by at meiosis I up to puberty with the beginning of ovulation, and conclude with fertilization.

Considering the differences between men and women in the regulation of meiosis in germ cells, it is postulated that women contribute more than men to aneuploidy rates due to non-chromosome disjunction of the homologous during meiosis I, with an increase in the frequency of errors in chromosome segregation with advancing age (1000 Genomes Project Consortium, 2010).

Hehir-Kwa *et al*. (2011) analyzed the parental origin analysis of 118 *de novo* CNVs using the CMA approach with a 250 K SNP matrix (Affymetrix, Santa Clara, USA). The study presented 76.3% (90/118) of paternal CNVs, and maternal CNVs represented 23.7% (28/118), showing that, in addition to *de novo* CNVs being mostly of paternal origin, it was also detected the rise of CNV with the increase of the parents’ age. These data do not corroborate with our study that demonstrated 60% of *de novo* pathogenic CNVs were of maternal origin. It is important to emphasize that the sample size of the present study can contribute randomly to this effect, which could correspond only to a sampling bias.

In our study, the parent origin of all 15 *de novo* pathogenic CNVs was analyzed, and we found 60% (9/15) derived from the maternal germ line and 40% (6/15) from the paternal germ line. Considering the gains in CNVs, we observed that 60% were paternal and 40% were maternal. On the other hand, mothers contributed more to losses CNVs, representing 70%, while the fathers contributed 30%. Some authors highlight that the contribution of point mutations and chromosomal rearrangements of *de novo* CNVs patients with intellectual disability are frequently from paternal origin, especially loss CNVs. However, we observed in our study that maternal CNVs were more prevalent (Ma et al., 2017; Hehir-Kwa et al., 2011; Thomas et al., 2006).

The meiotic division process of male germ cells requires multiple cell divisions to maintain the viable number of gametes for reproduction. This process associated with advanced paternal age could contribute to SNP accumulation or insertion/deletion mutations (indels) (Hehir-Kwa et al., 2011; Ma et al., 2017). According to Hehir-Kwa *et al.* (2011), the advanced paternal age impacts the formation of rare *de novo* CNVs. An Icelandic study showed that paternal age is a dominant factor in determining the number of *de novo* mutations in the child, where considering paternal age at conception, they observed an increase of about two mutations per year (Kong et al., 2012). On the other hand, Buizer-Voskamp et al. (2013) studied a larger cohort of healthy subjects and showed no paternal bias and age effect on global CNV burden. In our study, we observed the paternal contribution to the majority of non-recurrent CNVs, and we did not have evidence of a significant relation between the increase in CNV formation and the paternal age.

We observed that losses were twice as frequent as gains, corroborating worldwide findings reported by Cooper et al. (2011). It occurs because deletions can arise from crossovers in both *cis* and *trans*, whereas duplications can only occur through crossovers in *trans*. Besides, in pathogenic CNVs the impact on phenotypic tends to be more significant for deletions than duplications (Liu et al., 2012).

The three main LCR characteristics that contribute positively to genomic instability, favoring DNA rearrangements via the nonallelic homologous recombination (NAHR) process are (1) LCRs ≥ 10 kb in length, considered large LCRs, (2) the distance between LCRs of approximately 10 Mb, and (3) ≥ 97% sequence identity (Carvalho and Lupski, 2016; Harel and Lupski, 2018). In the present study, we analyzed the LCRs sequence identity and observed that 6/7 recurrent CNVs (85.7%) harboring LCRs with sequences similarity ≥ 98%.

The recurrent CNVs share breakpoints, genomic content, and size in unrelated individuals, and their mechanism of formation often occurs from NAHR mediated by directly oriented or inverted LCRs with a high identity that flank unique genome (Harel and Lupski, 2018). Ma et al. (2017) analyzed the presence of LCRs flanking the CNVs and revealed 65.5% (57/87) of recurrent CNVs, different from our study. On the other hand, Hehir-Kwa et al. (2011) analyzed 118 CNVs and observed that 25 CNVs were flanked by LCRs, representing 21.2%. In the current study, the presence of CNVs flanked by LCRs was observed in 46.7%. Thus, they were classified as recurrent CNVs formed by the NAHR process, which is one of the first mechanisms identified as responsible for the formation of genomic disorders (Lupski, 1998; Stankiewicz and Lupski, 2002; Harel and Lupski, 2018).

Non-recurrent CNVs have unique genomic content and size in unrelated individuals, and these non-recurrent CNVs are formed by different mechanisms that do not require LCR flanking the genome content, such as the nonhomologous end joining (NHEJ) mechanism and replication-based mechanisms (RBMs), highlighting the microhomology-mediated break-induced replication (MMBIR) and fork stalling and template switching (FoSTeS) (Harel and Lupski, 2018). In our study, we observed 53.3% (8/15) of CNVs that were not flanked by LCR with a similarity greater than 90%, and they were classified as non-recurrent.

Hehir-Kwa et al. (2011) suggested that the non-recurrent CNVs were generally paternal origin and generated by NHEJ and RBMs. Furthermore, because they are rare CNVs, they are distributed throughout the genome. These data corroborate with what we observed where of the six paternal CNVs identified, four are non-recurrent CNVs, representing 66.7%. Thus, we also confirmed that the formation mechanism of these CNVs with paternal origin was probably by NHEJ, FoSTeS, or MMBIR, which occurs frequently in spermatogonia that perform several mitotic divisions to increase the production of gametes. 

Overall, neurodevelopmental disorders, especially intellectual disability, are associated with rare recurrent CNVs in specific chromosome regions, and the majority of these CNVs have incomplete penetrance and variable expressivity influenced by other genetic and environmental factors (Torres et al., 2016; Carvalho and Lupski, 2016). In our study, we observed that of the 15 *de novo* pathogenic CNVs, most were of maternal origin. Also, we identified a large number of non-recurrent CNVs, the majority of which were of paternal origin. 

Compared with previous studies, we observed that our findings differed from some of these studies, which could be due to the composition of our cohort. Therefore, more studies that assess the parental origin of *de novo* pathogenic CNVs related to neurodevelopmental disorders to understand their formation mechanisms should be done because of their importance in understanding the role of events not mediated by NAHR in rearrangement formation in patients with intellectual disability.
